# A Novel Approach of Using Ground CNTs as the Carbon Source to Fabricate Uniformly Distributed Nano-Sized TiC_x_/2009Al Composites

**DOI:** 10.3390/ma8125495

**Published:** 2015-12-17

**Authors:** Lei Wang, Feng Qiu, Licheng Ouyang, Huiyuan Wang, Min Zha, Shili Shu, Qinglong Zhao, Qichuan Jiang

**Affiliations:** 1 Key Laboratory of Automobile Materials, Ministry of Education, and Department of Materials Science and Engineering, Jilin University, No. 5988 Renmin Street, Changchun 130025, China; leiwang798@163.com (L.W.); 18844562051@163.com (L.O.); wanghuyaun@jlu.edu.cn (H.W.); xunai85@126.com (M.Z.); zhaoqinglong@jlu.edu.cn (Q.Z.); 2 State Key Laboratory of Luminescence and Applications, Changchun Institute of Optics, Fine Mechanics and Physics, Chinese Academy of Sciences, Changchun 130012, China; shushili@ciomp.ac.cn

**Keywords:** Al composites, combustion synthesis, nano-sized TiC_x_, distribution, tensile properties

## Abstract

Nano-sized TiC_x_/2009Al composites (with 5, 7, and 9 vol% TiC_x_) were fabricated via the combustion synthesis of the 2009Al-Ti-CNTs system combined with vacuum hot pressing followed by hot extrusion. In the present study, CNTs were used as the carbon source to synthesize nano-sized TiC_x_ particles. An attempt was made to correlate the effect of ground CNTs by milling and the distribution of synthesized nano-sized TiC_x_ particles in 2009Al as well as the tensile properties of nano-sized TiC_x_/2009Al composites. Microstructure analysis showed that when ground CNTs were used, the synthesized nano-sized TiC_x_ particles dispersed more uniformly in the 2009Al matrix. Moreover, when 2 h-milled CNTs were used, the 5, 7, and 9 vol% nano-sized TiC_x_/2009Al composites had the highest tensile properties, especially, the 9 vol% nano-sized TiC_x_/2009Al composites. The results offered a new approach to improve the distribution of *in situ* nano-sized TiC_x_ particles and tensile properties of composites.

## 1. Introduction

Recent investigations have found that nano-particles can significantly increase the strength of the alloy matrix as compared to their micron-sized correspondents. For example, by adding a small percentage (1 vol%) of nano-sized Si_3_N_4_ (10 nm) particles, the tensile properties of Al matrix composites are similar to those of 15 vol% SiC_p_ (3.5 μm)/Al composites [1]. Several methods for preparing nano-sized particle reinforced metal matrix composites (MMCs) by using external addition methods have been reported, including powder metallurgy [1,2], mechanical alloying [3,4,5] and casting [6,7,8,9]. The drawbacks of these methods are that the reinforcing particles are susceptible to contamination and difficult to disperse in the alloy matrix [10]. In order to overcome these shortcomings, *in situ* methods, e.g., the molten salt, the combustion synthesis, have been developed to prepare the MMCs [11,12]. In contrast to the molten salt, the combustion synthesis takes advantage of the low energy requirement, less interfacial defects, a one step forming process, the dense and high purity of the products, and has drawn a lot of attention [13].

TiC is thermally a very stable refractory metal carbide and possesses high hardness. Many efforts have been made to fabricate TiC_x_ particle reinforced aluminum matrix composites (AMCs) using the combustion synthesis [14]. However, the TiC_x_ particles obtained are typically micron sized [12,15]. Studies on the combustion synthesis of nano-sized TiC_x_ particles are very rare. Actually, nano-sized TiC_x_ particles often exist in clusters in the composites due to their high surface energy, which more or less limits their industrial applications. Substantial work has been focused on the distribution of nano-sized TiC_x_ particles in AMCs. Very recently, Kim *et al.* [16] adopted the method of hybridization of nano-sized TiC particles with multi-walled carbon nanotubes (MWCNTs, 0.7 vol%) to improve the distribution of nano-sized TiC particles in the Al matrix. The results showed that MWCNTs could be used as carriers to improve the dispersion of nano-sized particles in AMCs. Jafarian *et al.* [17] fabricated nano-sized TiC/Al composites by accumulative roll bonding, where the distribution of nano-sized TiC was significantly improved. Also, Nemati *et al.* [18] studied the manufacture of nano-sized TiC/Al-4.5 wt% Cu composites produced by mechanical milling, where a reasonably uniform distribution of nano-sized TiC particles was observed. As reported above, it can be seen that, these studies mainly focus on the example of *ex situ* nano-sized TiC_x_ particles [6,16,17,18,19]. Researches on the distribution of *in situ* nano-sized TiC_x_ particles have rarely been reported.

In our previous work, by taking advantage of the finer size and high chemical activity of CNTs, nano-sized TiC_x_ particles of 20–60 nm could be synthesized by self-propagating high temperature synthesis (SHS) in Al-Ti-CNTs systems [20,21]. Based on this work, nano-sized TiC_x_/2009Al composites with 10–30 vol% TiC_x_ were successfully fabricated via the combustion synthesis combined with vacuum hot pressing followed by hot extrusion [22]. However, although the distribution of nano-sized TiC_x_ particles improved in the matrix after hot extrusion, aggregation of the nano-sized TiC_x_ particles still exists and the increase in tensile strength of the composites is not obvious. Moreover, we also found that the dispersion of CNTs in the Al-Ti-CNTs system is a key factor to determine the distribution of the synthesized nano-sized TiC_x_ in matrix. The fact that CNTs are usually entangled and twisted together leads to the formation of clusters of nano-sized TiC_x_ particles [23,24]. Therefore, research on correlating the effect of dispersion of CNTs and the distribution of synthesized nano-sized TiC_x_ particles as well as the tensile properties of nano-sized TiC_x_ reinforced AMCs is essential.

In this paper, the lower volume fraction (5 ,7, and 9%) nano-sized TiC_x_/2009Al composites were prepared via the combustion synthesis combined with vacuum hot pressing followed by hot extrusion. A novel approach to improve the distribution of synthesized nano-sized TiC_x_ particles in the matrix, where ground CNTs are used, is proposed. Meanwhile, the effects of dispersion of CNTs in the 2009Al-Ti-CNTs system on the distribution of nano-sized TiC_x_ particles, the microstructures, and the tensile properties of nano-sized TiC_x_/2009Al composites were investigated.

## 2. Experimental Section

The raw materials were Ti powders (>99.5% purity, ~48 μm), CNTs (10 to 20 nm in diameter and 20 to 100 μm in length) and 2009Al alloy powders (~75 μm). The chemical composition (in wt%) of 2009Al consisted mainly of 3.7 Cu, 1.3 Mg, 0.25 Si, 0.05 Fe and the balance with Al. Powder metallurgy 2009Al was selected as a comparative sample. CNTs were first milled alone for times varying from 1 h to 3 h in a cylindrical stainless steel drum. The rotational speed was 300 rad/min. Then, CNTs and Ti powders with a molar ratio of 1:1 were mixed with 2009Al alloy powders. The reactants, with nominal compositions of 91, 93, and 95 vol% 2009Al alloy powders, respectively, were mixed sufficiently by ball milling for 48 h and then condensed into cylindrical compacts (Φ 45 mm × 35 mm) with green densities of ~65% ± 2% of theoretical density, as with 2009Al alloy powders. The combustion synthesis and vacuum hot pressing experiments were conducted in a self-made vacuum furnace. The reactants were put into the furnace and then heated in a vacuum atmosphere. During the process, the temperature was monitored by W5-Re26 thermocouples. When the temperature measured by the thermocouple rose rapidly, indicating that the sample should be ignited, the sample was quickly pressed while it was still hot and soft.

The 2009Al and hot pressed composites were extruded at 773 K under an extrusion ratio of 19:1. Then the as-extruded composites were solution treated at 783 K for 1 h, followed by water quenching and aging at room temperature for 96 h.

The phase constitution of the synthesized composites were characterized by X-ray diffractometry (XRD, Rigaku D/Max 2500PC, Tokyo, Japan) with Cu Kα radiation utilizing a scanning speed of 4°/min. The distribution of TiC_x_ particles in as-extruded composites was observed using a field emission scanning electron microscope (FESEM, JSM 6700F, Tokyo, Japan) and transmission electron microscopy (TEM, JEM 2100F, Tokyo, Japan). The tensile fracture surface of the samples was examined by scanning electron microscope (SEM, Evo18, Carl Zeiss, Oberkochen, Germany). The mechanical property tests were carried out with a servo hydraulic materials testing system (MTS, MTS 810, Minneapolis, MN, USA) at a strain rate of 3 × 10^−4^ s^−1^.

## 3. Results and Discussion

### 3.1. Morphology Evolution of CNTs

**Figure 1:**
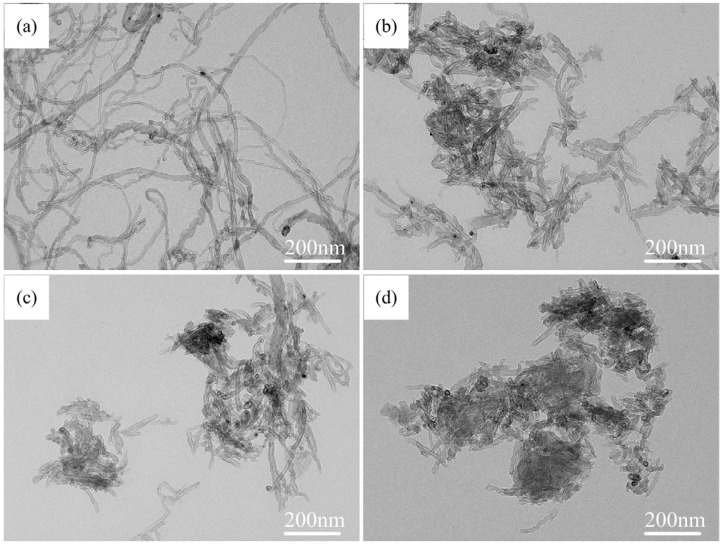
Transmission electron microscopy (TEM) images of (**a**) untreated CNTs and CNTs milled for (**b**) 1 h; (**c**) 2 h and (**d**) 3 h.

TEM images of CNTs are shown in Figure 1. Before treatment, the CNTs are entangled and twisted together with a diameter of 10–20 nm and length in the range of 20–100 μm, as shown in Figure 1a. After ball-milling, the long CNTs can be fragmented and shortened. From Figure 1b–d, one can see that the lengths of CNTs become shorter with increasing ball-milling time. After milling for 1 h, 2 h, and 3 h, the lengths of the CNTs decrease to ~500 nm, ~200 nm, and ~100 nm, respectively. Meanwhile, the specific surface area of the CNTs is increased by ball-milling as well. The shortened CNTs dissolve more rapidly in the liquid Al, which promotes the combustion synthesis reaction [21]. However, the CNTs became agglomerated because of the friction of rolling between the balls with increasing ball-milling time; the agglomeration of CNTs begins to become severe when CNTs are milled for 3 h. The effects of the agglomeration of CNTs on the distribution of the synthesized nano-sized TiC_x_ is discussed later.

### 3.2. Phase Constituents and Microstructures

**Figure 2:**
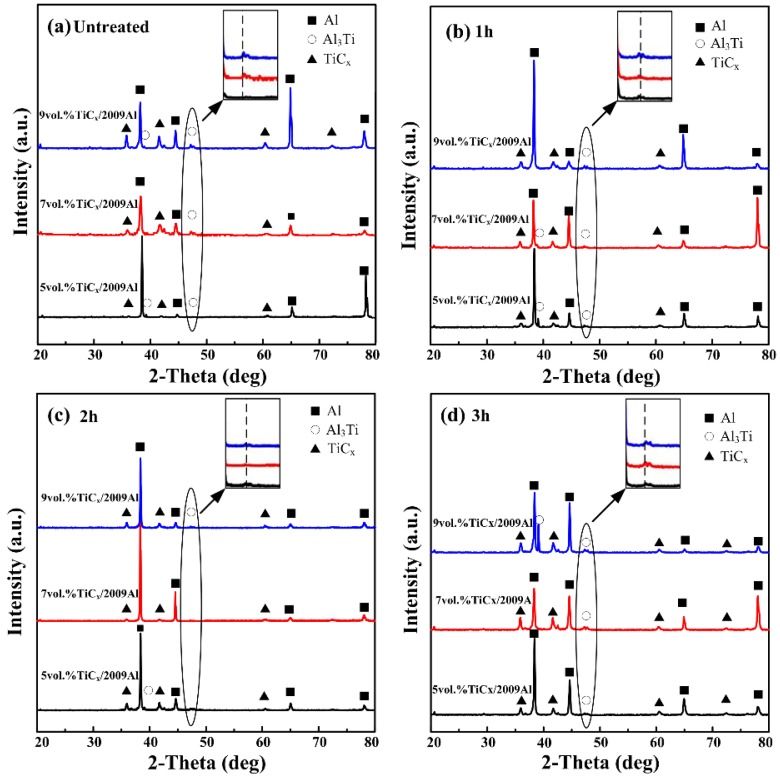
X-ray diffractometry (XRD) patterns of the nano-sized TiC_x_/2009Al composites prepared by using (**a**) untreated CNTs and CNTs milled for (**b**) 1 h; (**c**) 2 h and (**d**) 3 h as the carbon source.

Figure 2 shows XRD patterns of the 5, 7, and 9 vol% nano-sized TiC_x_/2009Al composites fabricated by using untreated CNTs and CNTs milled for 1 h, 2 h, and 3 h as the carbon source. It can be seen that in addition to the main phase constitutions, *i.e.*, TiC_x_ and α-Al phase, a small amount of Al_3_Ti phase is also detected. As indicated in the inset in Figure 2a–d, the (200) peak intensity of Al_3_Ti is obviously different. For CNTs milled for 1 h and 2 h, with increasing ball-milling time of CNTs, the (200) peak intensity of Al_3_Ti in the composites decreases as compared to untreated CNTs. However, when the 3 h-milled CNTs are used, the (200) peak intensity of Al_3_Ti in the composites tends to be similar to the untreated CNTs. It has been found that when the Al content in an Al-Ti-C system is high (≥80 wt%), TiC_x_ formation reaction tends to be incomplete and the intermediate Al_3_Ti phase is generated [21]. However, the high specific surface area of shortened CNTs leads to an increase in the contact area between CNTs and Al-Ti binary liquid phase. Meanwhile, an increase in the surface activity leads to an easy dissolution of CNTs into the Al-Ti binary liquid phase, promoting the combustion synthesis reaction. As a result, the amount of residual Al_3_Ti phase decreases in the final products. When shortened CNTs re-aggregate, the contact area between CNTs and the Al-Ti binary liquid phase and the dissolution rate of CNTs decreases, leading to an incomplete reaction in the Al-Ti-C system and an increase in the peak intensity of Al_3_Ti.

**Figure 3:**
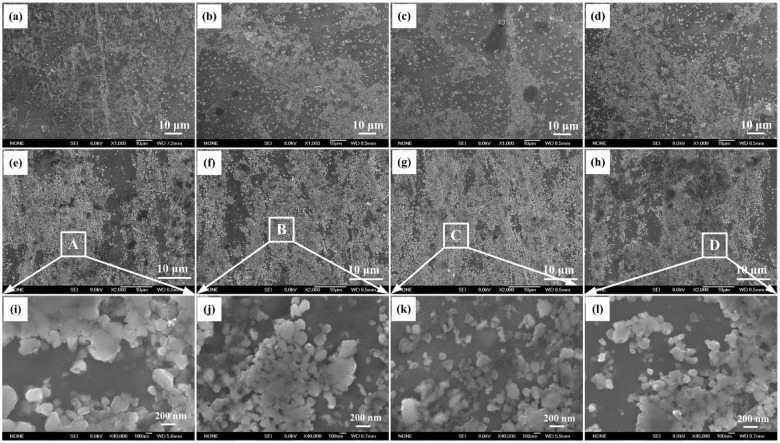
Field emission scanning electron microscope (FESEM) images of the synthesized nano-sized TiC_x_/2009Al composites fabricated by using (**a**) untreated CNTs and CNTs milled for (**b**) 1 h; (**c**) 2 h and (**d**) 3 h as the carbon source; (**e**–**h**) the corresponding as extruded samples and (**i**–**l**) the magnification of FESEM images of areas A, B, C and D in the corresponding composites.

Figure 3 shows FESEM images of the synthesized nano-sized TiC_x_/2009Al composites fabricated by using untreated CNTs and CNTs milled for 1 h, 2 h, and 3 h as the carbon source. As shownin Figure 3a–d, it can be observed that the microstructure consists of two different zones: a thick zone with a high density of TiC_x_ particles, alternated with the other with a relatively low density of TiC_x_ particles filling the spacing between the thick ones. By using ground CNTs, the thick zone with a high density of TiC_x_ particles seems to decrease. After hot extrusion, with increasing ball-milling time of CNTs from 1 h to 2 h, the dispersion of synthesized nano-sized TiC_x_ particles improved obviously. However, with further increasing milling time to 3 h, the synthesized nano-sized TiC_x_ particles seem to become re-aggregated, as shown in Figure 3e–h. To further study the effect of ground CNTs on the distribution of synthesized TiC_x_ particles in the aluminum matrix, the high magnification of A, B, C, and D areas in the corresponding as extruded samples were researched. As showed in Figure 3i–l, when CNTs milled for 2 h are used, the nano-sized TiC_x_ clusters are well separated. The observations so far demonstrated that the present method for the production of nano-sized TiC_x_/2009Al composites is capable of providing a relatively uniform nano-sized TiC_x_ dispersion.

### 3.3. Tensile Test and Fracture Surface Study

**Figure 4:**
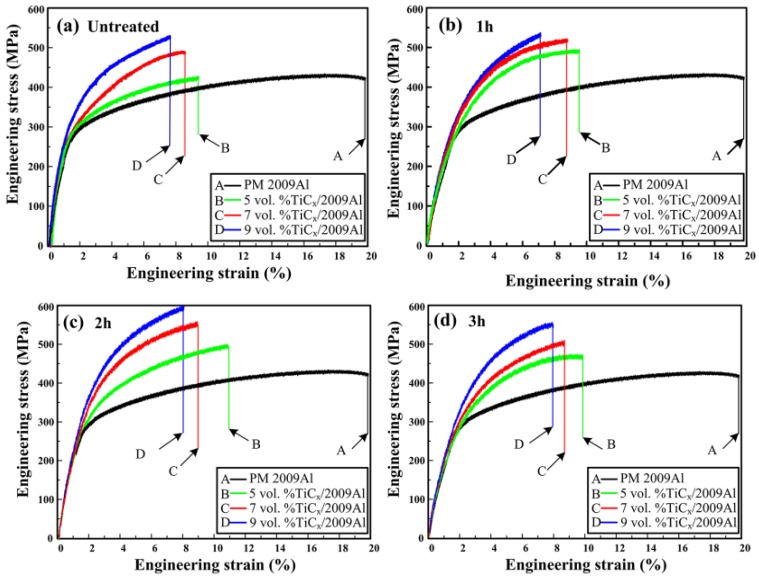
Engineering stress-strain curves of PM 2009Al and 5, 7, 9 vol% nano-sized TiC_x_/2009Al composites prepared by using (**a**) untreated CNTs and CNTs milled for (**b**) 1 h; (**c**) 2 h and (**d**) 3 h as the carbon source.

Figure 4a–d shows engineering stress-strain curves of PM 2009Al and the 5, 7, and 9 vol% nano-sized TiC_x_/2009Al composites fabricated by using untreated CNTs and CNTs milled for 1 h, 2 h, and 3 h as carbon source. The tensile properties of 2009Al and all the composites are listed in Table 1 and Table 2. Clearly, the strength of 2009Al can be enhanced by nano-sized TiC_x_ particles. When CNTs milled for 1 h, 2 h, and 3 h are used, the yield strength (σ_0.2_), tensile strength (σ_b_) and fracture strain (ε_f_) of the investigated TiC_x_/2009Al composites increased dramatically. Among all the investigated composites, the ones fabricated by using 2 h-milled CNTs exhibit the highest yield strength, tensile strength, and fracture strain. Also, the tensile strength of the composites increases with increasing volume fraction of nano-sized TiC_x_ particles. Especially, the 9 vol% nano-sized TiC_x_/2009Al possesses superior tensile properties, e.g., yield strength, tensile strength, and fracture strain of ~404 MPa, ~601 MPa, and ~10.8%, respectively, which increased by ~19.5%, ~15.8%, and ~5.2%, respectively, as compared to the composites fabricated by using untreated CNTs.

**Table 1:** The tensile test data of 2009Al.

Samples	σ_0.2_/MPa	σ_b_/MPa	ε_f_/%
PM2009Al	269${}_{-6}^{+7}$	441${}_{-8}^{+3}$	19.8${}_{-1.7}^{+0.9}$

**Table 2:** Room temperature tensile properties of 5, 7, and 9 vol% TiC_x_/2009Al composites with (**a**) 0 h, (**b**) 1 h, (**c**) 2 h and (**d**) 3 h ball milling pretreated carbon nanotubes.

Times	5 vol% TiC_X_/2009Al			7 vol% TiC_X_/2009Al			9 vol% TiC_X_/2009Al		
	σ_0.2_/MPa	σ_b_/MPa	ε_f_/%	σ_0.2_/MPa	σ_b_/MPa	ε_f_/%	σ_0.2_/MPa	σ_b_/MPa	ε_f_/%
0 h	276${}_{-14}^{+11}$	415${}_{-10}^{+5}$	9.3${}_{-0.8}^{+1.1}$	303${}_{-7}^{+6}$	497${}_{-10}^{+4}$	8.4${}_{-0.8}^{+1.5}$	338${}_{-9}^{+10}$	519${}_{-6}^{+2}$	7.7${}_{-0.6}^{+0.5}$
1 h	289${}_{-7}^{+8}$	496${}_{-3}^{+2}$	9.6${}_{-1.0}^{+0.7}$	323${}_{-10}^{+3}$	516${}_{-4}^{+3}$	8.8${}_{-1.2}^{+0.7}$	349${}_{-6}^{+7}$	535${}_{-6}^{+4}$	7.2${}_{-1.0}^{+0.7}$
2 h	315${}_{-5}^{+10}$	509${}_{-7}^{+8}$	10.8${}_{-1.3}^{+0.9}$	359${}_{-9}^{+5}$	553${}_{-11}^{+8}$	9.0${}_{-0.9}^{+0.3}$	404${}_{-11}^{+6}$	601${}_{-7}^{+1}$	8.1${}_{-1.5}^{+0.9}$
3 h	287${}_{-6}^{+7}$	461${}_{-5}^{+3}$	9.9${}_{-0.4}^{+0.5}$	313${}_{-6}^{+8}$	504${}_{-6}^{+3}$	8.6${}_{-1.3}^{+1.1}$	331${}_{-5}^{+7}$	543${}_{-5}^{+2}$	7.9${}_{-1.4}^{+1.1}$

**Figure 5:**
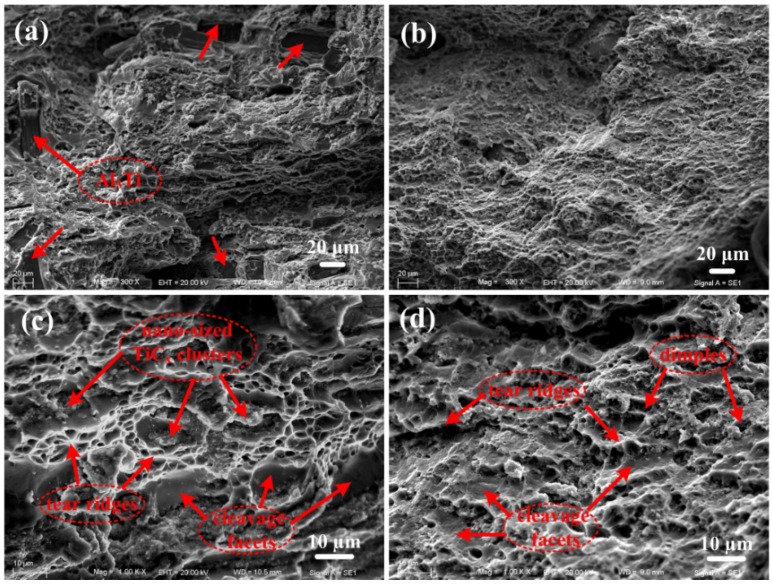
Scanning electron microscope (SEM) images of the tensile fracture surface of 9 vol% nano-sized TiC_x_/2009Al composite fabricated by using (**a**) untreated CNTs; (**b**) CNTs milled for 2 h as carbon source; (**c**) and (**d**) are the high magnified images of the corresponding composites.

Figure 5 shows SEM images of the tensile fracture surface of 9 vol% nano-sized TiC_x_/2009Al composite fabricated by using (a) untreated CNTs; (b) CNTs milled for 2 h as carbon source. It can be observed that in contrast to the sample fabricated by using 2 h-milled CNTs, there exists some coarse Al_3_Ti with strip shape on the tensile fracture surface of the sample fabricated by using untreated CNTs. The coarse Al_3_Ti with strip shape decreased the tensile strength and ductility of the composites. Figure 5c–d shows the high magnified images of corresponding composites. One can see that the fracture surfaces exhibit a mixture of cleavage facets, tear ridges, small dimples, as well as local agglomeration of the nano-sized TiC_x_ particles. Compared with the sample fabricated by using untreated CNTs, more homogeneous dimples are obtained on the fracture surface of the sample fabricated by using 2 h-milled CNTs. Generally, matrix cracks are inclined to divert at the interface with the tensile loading involved. When the material is employed, local agglomeration of TiC_x_ particles cannot undertake stress transmission efficiently under a given load and results in brittle failure, which could degrade the ductility of the composite.

In addition, note that when 2 h-milled CNTs are used, the tensile strength of the 9 vol% nano-sized TiC_x_/2009Al composites, *i.e.*, ~601 MPa, is apparently higher than that of 15 vol% SiC_p_ (7 μm)/2009Al composites, *i.e.*, ~520 MPa, and that of 15 vol% SiC_p_ (5 μm)/2009Al composites, *i.e.*, ~536 MPa. Both of the latter are fabricated by powder metallurgy [25,26]. It is believed that the significant improvement in tensile properties should be attributed to the relatively uniform distribution of the nano-sized TiC_x_ particles induced by using ground CNTs as carbon source.

### 3.4. Interface between Nano-Sized TiC_x_ Particles and Al Matrix

**Figure 6:**
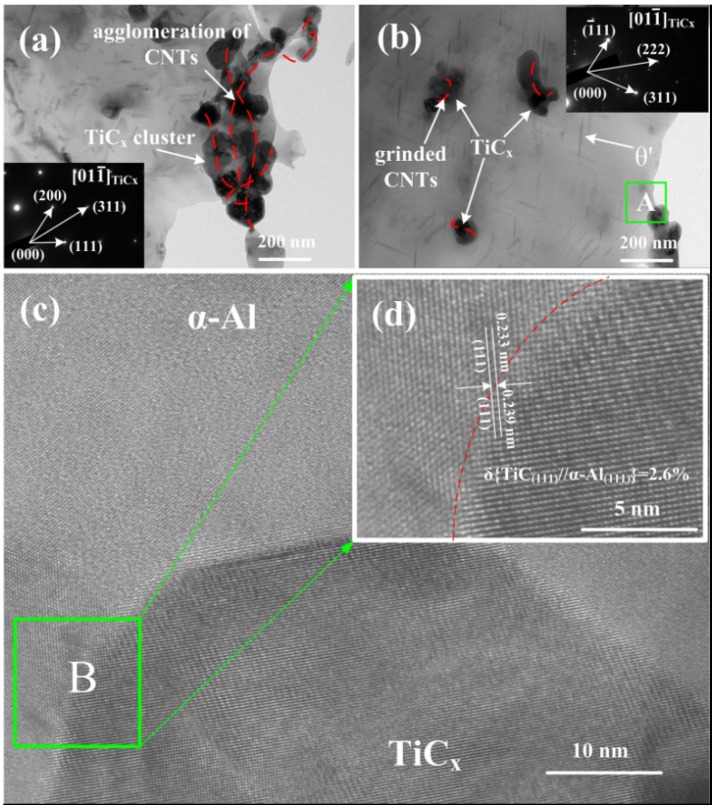
(**a**–**b**) TEM images of as-extruded 9 vol% TiC_x_/2009Al composites prepared by using (**a**–**b**) untreated CNTs and 2 h-milled CNTs as the carbon source, insert: selected-area electron diffraction (SAED) pattern of TiC_x_ particles; (**c**) HRTEM image of the area A in Figure 6b; (**d**) the magnification of HRTEM images of areas B.

TEM images of the as-extruded 9 vol% nano-sized TiC_x_/2009Al composites are shown in Figure 6. The selected-area electron diffraction (SAED) pattern in Figure 6a,b corresponds to the (01–1) zone axis of the TiC_x_ particle. Considering untreated CNTs are usually curved and twisted together, when it is used, C-rich regions will form in molten aluminum alloy. In these C-rich regions, the nano-sized TiC_x_ particles form and aggregate simultaneously (Figure 6a). However, the nano-sized TiC_x_ clusters seem to be broken up when 2 h-milled CNTs are used (Figure 6b). One can easily understand that a relatively uniform distribution of shortened CNTs in the Al-Ti-CNTs system leads to a better distribution of synthesized nano-sized TiC_x_. Figure 6c shows a HRTEM image of area A in Figure 6b, where a good interfacial bonding without cracks or apparent interfacial reaction between nano-sized TiC_x_ particles and α-Al matrix can be observed. Meanwhile, a small lattice mismatch (~2.6%) between nano-sized TiC_x_ and α-Al is observed in Figure 6d. As a result, *in situ* nano-sized TiC_x_/2009Al composites have a good interfacial bonding between nano-sized TiC_x_ and α-Al matrix, as compared to the composites processed by *ex situ* methods.

### 3.5. Mechanism Analysis

**Figure 7:**
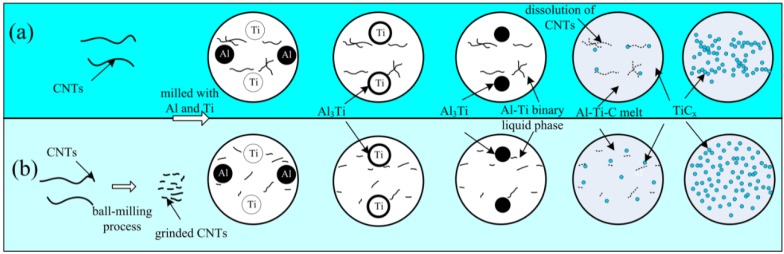
The proposed reaction mechanism of the combustion reaction by using (**a**) untreated CNTs; (**b**) ground CNTs as carbon source in the 2009Al-Ti-CNTs system.

Substantial research pertaining to the combustion reaction mechanism of the Al-Ti-C system has been reported, of which the reaction mechanism can be described with a reaction-dissolution-precipitation model [27,28,29]. In the present work, as shown in Figure 7a, Al firstly reacts with Ti to form Al_3_Ti around Ti particles in the 2009Al-Ti-CNTs system. With increasing temperature, Al_3_Ti melts to form an Al-Ti binary liquid phase, meanwhile, CNTs dissolve in the Al-Ti binary liquid phase to form an Al-Ti-C ternary liquid phase. When the concentration of [Ti] and [C] in the Al-Ti-C ternary liquid phase is high enough for reactions between [Ti] and [C] to occur, TiC_x_ will be synthesized and precipitate out of the melts. The reaction will release a lot of heat, accompanying the rapid dissolution of CNTs in the Al-Ti-C ternary liquid phase, and meanwhile, promoting the combustion synthesis reaction until the reaction ends. When CNT powder becomes aggregated, C-rich regions tend to form, where TiC_x_ particles precipitate out as agglomerates (Figure 6a). After ball milling of CNTs, as shown in Figure 7b, the much finer CNTs enlarge the area of the contact surface between the CNTs and the Al-Ti binary liquid phase, which accelerate the dissolution of CNTs in the Al-Ti-C ternary liquid phase and promote the combustion synthesis reaction. Meanwhile, the more evenly distributed CNTs decrease the amount of C-rich regions. Finally, nano-sized TiC_x_ becomes relatively uniformly distributed.

It has been found that the strength of composites is greatly influenced by the spatial distribution of particles [30,31]. When the second phase particles have a very small size and become more uniformly dispersed, it is difficult for dislocations to cut through or over. In the present study, the nano-sized TiC_x_ particle clusters usually act as crack sources as well as path of crack propagation, leading to the fracture of composites. Also, the Orowan strengthening effect of nano-sized TiC_x_ particles on the matrix is weakened as well due to these agglomerations. However, by using ground CNTs as carbon source, nano-sized TiC_x_ becomes relatively uniformly distributed. Less agglomeration of nano-sized TiC_x_ particles decreases the amount of crack sources as well as the paths of crack propagation, but meanwhile increases the amount of nano-sized TiC_x_ particles available, where the movement of dislocations can be effectively impeded. As a result, the tensile strength and fracture strain of the nano-sized TiC_x_/2009Al composites could be improved considerably by a more uniform distribution of the TiC_x_ particles due to the utilization of ground CNTs as carbon source.

## 4. Conclusions

The 5, 7, and 9 vol% nano-sized TiC_x_/2009Al composites were successfully fabricated by the combination of combustion synthesis and vacuum hot pressing followed by hot extrusion. When ground CNTs were used, the distribution of synthesized nano-sized TiC_x_ particles in the 2009Al matrix was dramatically improved, especially for the composites fabricated by using 2 h-milled CNTs. Tensile testing showed that when 2 h-milled CNTs were used, the 9 vol% nano-sized TiC_x_/2009Al composites had a superior combination of a high yield strength , tensile strength, and fracture strain (~404 MPa, ~601 MPa, and ~10.8%), which increased by ~19.5%, ~15.8%, and ~5.2%, respectively, as compared to the composites fabricated by using untreated CNTs as carbon source. The simultaneous high tensile strength and fracture strain could be attributed to the relatively uniform dispersion of nano-sized TiC_x_ induced by using ground CNTs as carbon source.
